# Microvascular narrowing and BP monitoring: A single centre observational study

**DOI:** 10.1371/journal.pone.0210625

**Published:** 2019-03-14

**Authors:** Fariya Ali, Mark Tacey, Nick Lykopandis, Deb Colville, Ecosse Lamoureux, Tien Y. Wong, William Vangaal, Anastasia Hutchinson, Judy Savige

**Affiliations:** 1 The University of Melbourne Department of Medicine, Northern Health, Epping, Victoria, Australia; 2 Department of Cardiology, Northern Health, Epping, Victoria, Australia; 3 Centre for Eye Research Australia, The Royal Victorian Eye and Ear Hospital, East Melbourne, Victoria, Australia; GPR Klinikum, GERMANY

## Abstract

**Introduction:**

Half of all hypertensive individuals have inadequately-controlled BP because monitoring methods are ineffective. This single centre study examined consecutive subjects undergoing 24 hour BP measurements for clinic and ambulatory BP levels, and for end-organ damage (retinal microvascular abnormalities and left ventricular hypertrophy, LVH, > 1.1 cm). Retinal images were graded for microvascular retinopathy (Wong and Mitchell classification), and vessel calibre using a semiautomated method. Features were compared using chi-squared, Fisher’s exact or the student’s t test.

**Methods:**

One hundred and thirty-one individuals (59 male, 45.0%, mean age 61.7 ± 14.5 years) were studied. Ninety-nine (76.2%) had a clinic BP ≥ 140/90 mm Hg, 84 (64.6%) had a mean awake systolic BP ≥ 135 mm Hg, 100 (76.9%) had a mean sleeping systolic BP ≥ 120 mm Hg, and 100 (76.2%) had abnormal nocturnal BP dipping patterns. Sixty-nine individuals had undergone echocardiography and 23 (33.3%) had LVH.

**Results:**

All participants had a mild (88.5%) or moderate (11.5%) microvascular retinopathy. Moderate microvascular retinopathy was found in 86.7% of those with a mean awake systolic BP ≥135 mm Hg (p = 0.058) but was not associated with other abnormal BP measurements, abnormal dipping patterns or LVH. However retinal arteriole calibre was reduced in subjects with a mean 24 hour awake systolic BP ≥ 135 mm Hg (p = 0.05). Retinal arteriole calibre was smaller in subjects with LVH (128.1 ± 13.5 μm compared with 137.6 ± 14.1 μm in normals, p = 0.014). Venular calibre was also less in subjects with LVH (185.4 ± 24.6 μm compared with 203.0 ± 27.2 μm in normals, p = 0.016). Arteriole narrowing predicted an increased risk of LVH (AUC 0.69, 95%CI 0.55 to 0.83) that was comparable with 24 hour systolic BP ≥130 mm Hg (AUC 0.68, 95%CI 0.53 to 0.82) and mean awake systolic BP ≥135 mm Hg (AUC 0.68, 95%CI 0.54 to 0.83).

**Conclusions:**

This study suggests that retinal arteriole narrowing may be equally accurate in predicting LVH as any clinic or ambulatory BP measurement. The convenience and accuracy of microvascular calibre measurement mean that it should be investigated further for a role in routine hypertension assessment and monitoring.

## Introduction

Hypertension affects one billion people worldwide, and is a major preventable cause of premature death and disability [1]. However hypertension is poorly controlled in up to half of all treated patients [1,2], and suboptimal control is also associated with higher rates of cardiac morbidity and mortality [3].

BP is commonly assessed in the clinic, at home by self-monitoring, by ambulatory or 24 hour BP measurement, and from end-organ damage. Other measures, such as vascular stiffness and pulse wave velocity are research tools and not widely available [4]. Hypertension in any setting, in the clinic, at home or with ambulatory monitoring, is associated with increased cardiac risk [5]. Even mild hypertension increases the risk of cardiovascular disease, stroke and renal failure. Some features measured on ambulatory BP monitoring, such as mean systolic BP, mean awake systolic BP, and the variations with sleep (non-dippers, reverse dippers and extreme dippers) are associated with increased cardiac risk [6].

NICE advocates that any individual with two clinic BP readings ≥140/90 mm Hg should undergo ambulatory BP monitoring, based on its accuracy and economic advantages, and correlation with cardiac risk [7,8]. Elevations predict an increased likelihood of left ventricular hypertrophy (LVH), microalbuminuria and stroke, and cerebral white matter changes [9], and the lowering of ambulatory BP with antihypertensive treatment correlates with LVH regression [10].

However ambulatory BP monitoring underestimates the BP level [11] and has issues with reproducibility as well as other limitations [12]. It is not suitable for assessment in the primary care setting nor for repeated measurement, for example, at monthly intervals. Measurements are real- time and do not reflect the BP over the previous weeks or months. Monitoring is performed only during weekdays when the BP is typically higher. Testing is inconvenient, since subjects have to attend hospital on consecutive days to have the device fitted, and some cannot tolerate the night-time measurements [13]. The technique is expensive to operate, most hospitals have only a few machines so there is frequently a delay in testing and ambulatory BP readings cannot be used for mass screening or epidemiological surveys. Some health systems only reimburse testing where white coat hypertension is suspected or not at all.

Poorly-controlled BP probably does not result from a lack of effective antihypertensive agents, but rather from poor monitoring techniques. The clinic reading underestimates the BP level. Self-monitoring requires repeated measurements, and duplication of equipment. Ambulatory BP monitoring is not suitable for routine and repeated testing. BP is a continuous variable and any single measurement must be a surrogate for BP readings over a period of time. Thus poor BP control may be assessed more accurately by the demonstration of end-organ damage.

End-organ damage is an early marker of clinical worsening of hypertension, and can be used to assess the efficacy of antihypertensive treatment over time. The main sites of damage are the heart, and the vascular system. Hypertension results in a microvascular retinopathy and narrowed vessels can potentially be used to measure end-organ damage and indirectly BP control. Microvascular disease includes generalised arteriolar narrowing, haemorrhage, and cotton wool spots [14,15]. Assessing retinal small vessel damage is fast, reproducible, inexpensive, convenient, non-invasive, and suitable for routine and repeated testing in a primary care or hospital setting. Already, retinal photography is available in suburban shopping centres to screen for diabetic complications and macular degeneration.

Thus, this study examined individuals undergoing BP monitoring, for a relationship between microvascular retinopathy including arteriole narrowing, and hypertension demonstrated by clinic or ambulatory BP, abnormal nocturnal dipping patterns and LVH.

## Study design and participants

### Study design

This was a single centre, cross-sectional, observational study of Caucasian subjects undergoing assessment of hypertension, with clinic and ambulatory BP measurements, including assessment of nocturnal dipping patterns. They were also evaluated for hypertensive end-organ damage, in the form of microvascular retinopathy and LVH where echocardiography was available within the previous 6 months.

Subjects were recruited consecutively from a Melbourne metropolitan teaching hospital (Northern Health) over a 6 month period. Recruitment, data capture, BP measurements and retinal imaging were coordinated in a single episode. Participants were interviewed at the time of ambulatory BP testing for clinical indications. They were assisted by the researcher to complete a structured questionnaire, and underwent retinal photography. Images were used to assess microvascular damage and vessel calibre using standardised protocols by trained graders. There were no changes to the study design after its commencement and no interim analyses.

The primary outcome was to demonstrate a relationship between microvascular retinopathy including arteriole narrowing, and hypertension demonstrated by clinic or ambulatory hypertension, abnormal nocturnal dipping patterns and LVH.

This study was approved by the Northern Health Human Research Ethics Committee according of the Principles of Helsinki, and all participants provided signed, informed consent.

### Study subjects

Subjects were recruited consecutively immediately prior to testing in an ambulatory BP laboratory after referral for clinical indications. Exclusion criteria were age under 18 years; incomplete ambulatory BP monitoring; asymmetric septal hypertrophy (ASH) and hypertrophic cardiomyopathy (HOCM) on echocardiography; and ungradable retinal images. Subjects were recruited regardless of whether their hypertension was newly diagnosed or treated, and its duration was not assessed. Randomisation was not performed.

### Measurement

Subjects were assisted to complete a structured questionnaire for demographics (age, gender, ethnicity), and vascular risk factors (smoking, diabetes, hypertension, dyslipidemia). Generally the diagnoses of hypertension, diabetes, and dyslipidemia were based on self-reported physician-made diagnoses. Current medications were extracted from individual electronic medical records, and the number of antihypertensive medications counted for each participant by a physician.

Participants were rested and a clinic BP measured using a Hg sphygmomanometer by a trained scientist. Clinic hypertension was defined as ≥ 140 /90 mm Hg.

Ambulatory BP monitoring was performed according to a standard protocol by the same laboratory scientist. The monitor (Tonoport V) was pre-set to a 24 hour cycle. Daytime readings were measured every 30 minutes during the day from 1 pm until 6 pm. From 6 pm to 5 am the following day, night time BP were recorded. After 10 pm the monitor measured BP on an hourly basis and the recording ceased at 5 am the next day. Measurements taken by the BP monitor were calculated to an average systolic and diastolic BP, and mean awake and sleeping BP.

For ambulatory BP monitoring, hypertension was defined as ≥130/80 mm Hg over 24 hours, and ≥ 135/85 mm Hg for daytime, ≥ 120/75 mm Hg for night time [16]. Normally BP falls in the first few hours of sleep, and increases in the early morning in the transition to wakefulness [16]. Subjects with nocturnal decreases in BP were termed ‘dippers’ if their BP fell more than 10% of the day-night difference, ‘non-dippers’ if the nocturnal BP stayed within the normal range of 0–10%; ‘reverse dippers’ if the BP fell less than 0%; and ‘extreme dippers’ if the nocturnal BP fall was greater than 20% of the day-night difference [17].

#### Transthoracic echocardiography

Subjects who had undergone transthoracic echocardiography (Siemens Acuson SC-2000) for clinical indications in the previous 6 months in the Northern Health laboratory had their results assessed for interventricular septum and posterior wall thickness >1.1 cm by a cardiologist (WVG).

#### Retinal imaging, microvascular grading and caliber measurements

Subjects underwent retinal colour photography of both eyes using a non-mydriatic retinal camera (KOWA 7, Japan). Standard 45^o^ images were taken of both eyes, with at least one centred on the macula and another on the optic disc.

All images were de-identified and microvascular retinopathy was graded using the Wong and Mitchell classification [18] by two trained graders, independently. Results were then compared, and discussed until consensus was reached. Where there was no consensus, the opinion of the ophthalmologist was accepted. Mild retinopathy was characterised by generalised arteriolar narrowing (arteriolar width <67% venular width), focal arteriolar narrowing (< 2/3 of the venular width), silver wiring (highly refractile vessel walls), low arteriovenous ratio (<2/3) or any combination of these signs. Moderate retinopathy was characterised by haemorrhage, cotton wool spots, hard exudates, or a combination of these. Severe retinopathy was distinguished by optic disc swelling.

Retinal vessel calibre was measured by a trained grader at the Centre for Eye Research Australia (Victoria, Australia) used a standardized protocol for grading the digital retinal images [19,20]. All vessels passing through a zone 0.5–1 disc diameters from the optic disc margin were examined using a semi-automated computer imaging program (University of Wisconsin, WI), and measures based on the 6 largest vessels were combined into the Central Retinal Artery and Vein Equivalents (CRAE and CRVE), using a computer-assisted method and Knudtson’s modification of the Parr-Hubbard formula(19, 20). This method was highly reproducible with high intra-class correlation coefficients [17].

### Statistical analysis

Categorical variables were presented as frequencies and percentages, continuous normally-distributed data as mean and standard deviation, and non-normal continuous data as median and interquartile range. Chi-squared and Fisher’s exact tests were used to test for associations between categorical variables, and students’ t-tests for normal continuous data. Multivariable analysis was performed using logistic regression models, and adjusting for nocturnal variation, LVH, grading of retinopathy (mild or moderate), and retinal arteriolar and venular calibre where appropriate. Statistical analyses were performed using STATA version 15.1 (Stata Corp, College Station, TX, USA), and a p-value of less than 0.05 was used to indicate statistical significance.

## Results

### Clinical and BP characteristics

One hundred and fifty-five subjects were recruited. Twenty-four (15%) were excluded because of incomplete 24 hour BP monitoring (n = 16) or ungradable retinal images (n = 8). None had asymmetric septal hypertrophy or hypertrophic cardiomyopathy, resulting in a total of 131 (85%) subjects included in the study.

The subjects’ mean age was 61.7 ± 14.5 years, and 59 (45%) were male **(Table 1)(S1 Table)**. Forty-one (31%) had known cardiac disease. Forty (31%) had diabetes, and 19 (18%) were current or former smokers. Just more than half the subjects were taking at least one antihypertensive medication, with 25% (n = 33) being on three or more. Their main indications for ambulatory BP monitoring were poorly-controlled BP (n = 90, 69%), postural hypotension (16%), fluctuating BP (9%) or suspected white coat hypertension (6%).

**Table 1:** Clinical and BP characteristics of study subjects.

Characteristics of subjects (n = 131)	Number (%)
**Age (mean, SD, years)**	61.7, 14.5
**Gender (male)**	59 (45.0)
**Number of antihypertensive medications (mean, SD)**	1.4 (1.8)
**Diabetes**	40 (30.5)
**Smokers or former smokers**	19 (17.8)
**Indications for ambulatory BP monitoring**	
**• Poor BP control**	90 (68.7)
**• Postural hypotension**	21 (16.0)
**• ‘white coat’ hypertension**	8 (6.1)
**• Fluctuating BP**	12 (9.2)
**Clinic BP**	
**• BP ≥ 140/90**	99 (76.2)
**• Systolic BP ≥ 140 mmHg**	96 (73.8)
**• Diastolic BP (≥ 90 mmHg)**	50 (38.5)
**Ambulatory blood pressure characteristics**	
**Mean 24 hour BP**	
**• Systolic BP (≥ 130 mmHg)**	93 (71.5)
**• Diastolic BP (≥ 80 mmHg)**	84 (64.6)
**Mean awake BP**	
**• Systolic BP (≥ 135 mmHg)**	84 (64.6)
**• Diastolic BP (≥ 85 mmHg)**	69 (53.1)
**Mean sleeping BP**	
**• Systolic BP (≥ 120 mmHg)**	100 (76.9)
**• Diastolic BP (≥ 75 mmHg)**	71 (54.6)
**Nocturnal variation in BP**	
**• Dippers**	31 (23.7)
**• Extreme dippers**	7 (5.3)
**• Non dippers**	67 (51.1)
**• Reverse dippers**	26 (19.8)
**Left ventricular hypertrophy (n = 69)**	23 (33.0%)

Ninety-nine subjects (76%) had a clinic BP ≥ 140/90 mmHg, with 96 (74%) having a systolic BP ≥ 140 mmHg and 50 (39%) with a diastolic BP ≥ 90 mmHg **(Table 1)**. On ambulatory BP monitoring, 93 subjects (72%) had a mean 24 hour systolic BP ≥ 130 mmHg, and 84 (65%) had a diastolic BP ≥ 80 mmHg. Eighty-four (65%) subjects had a mean awake systolic BP ≥ 135 mmHg and 69 (53%) had a mean diastolic BP ≥ 85 mmHg. One hundred (77%) had a mean asleep systolic ≥ 120 mmHg and 71 (55%) had a sleeping diastolic BP ≥ 75 mmHg. Thirty-one (24%) subjects were classified as ‘dippers’, and 100 were ‘non-dippers’ or had an abnormal nocturnal BP dipping pattern. Seven (5%) were ‘extreme dippers’, 67 (51%) ‘non- dippers’ and 26 (20%) ‘reverse dippers’. Sixty-nine had had an echocardiogram, 23 (33%) of which demonstrated LVH.

### Retinal microvascular retinopathy

All 131 subjects (100%) had features of a microvascular retinopathy. Consensus was reached on grading severity between the two graders in all cases. Retinopathy was mild in 116 (89%) and moderate in 15 (11%). None had a severe retinopathy.

A higher proportion of subjects with moderate microvascular retinopathy had a mean awake systolic BP ≥ 135 mmHg (87%) when compared to those with mild microvascular retinopathy (62%), although this did not reach statistical significance 0.058). This was confirmed upon multivariable analysis (OR 4.43, 95% CI: 0.93–21.2, p = 0.062). There was no indication of any association between microvascular retinopathy and elevated clinic BP, or any other feature on ambulatory BP monitoring, nor LVH **(Table 2).**

**Table 2:** Microvascular retinopathy and BP characteristics.

BP Characteristic	Microvascular retinopathy		
	Mild	Moderate	p-value
Subjects	116	15	
Clinic systolic BP ≥ 140 mmHg	88 (76.5)	11(73.3)	0.79
Ambulatory BP monitoring			
Mean 24-hr Systolic BP ≥ 130 mmHg	80 (69.6)	13 (86.7)	0.17
Mean 24-hr Diastolic BP ≥ 80 mmHg	73 (63.5)	11 (73.3)	0.45
Mean 24-hr awake Systolic BP ≥ 135 mmHg	71 (61.7)	13 (86.7)	0.058
Mean 24-hr awake Diastolic BP ≥ 85 mmHg	59 (51.3)	10 (66.7)	0.26
Mean 24-hr asleep Systolic BP≥ 120 mmHg	88 (76.5)	12 (80.0)	0.76
Mean 24-hr asleep Diastolic BP≥ 75 mmHg	61 (53.0)	10 (66.7)	0.32
Nocturnal variation in BP			0.10
** **Dippers	30 (25.9)	1 (6.7)	
** **Other forms of Dipping (severe, reverse, non-dippers)	86 (74.1)	14 (93.3)	
LVH	19 (32.0)	4 (44.0)	0.45

### Left ventricular hypertrophy

Sixty-nine subjects (53%) had undergone echocardiography in the previous 6 months, of whom 23 (33%) had LVH. Clinic and ambulatory BP monitoring means were typically higher in those with LVH, but not significantly so. A higher proportion of subjects with LVH had a mean sleeping diastolic BP ≥ 75 mmHg (70%) compared with those with no LVH (44%) (p = 0.050). This maintained statistical significance after adjusting for confounding effects (p = 0.044). There was no association between moderate microvascular retinopathy and LVH (p = 0.45) or an abnormal dipping pattern and LVH (p = 1.00) **(Table 3)**.

**Table 3:** Left ventricular hypertrophy, and BP characteristics and retinopathy.

BP Characteristic	Left Ventricular Hypertrophy		
	No	Yes	p-value
Subjects	46	23	
Clinic BP ≥ 140/90 mmHg	29 (64)	19 (83)	0.12
Ambulatory BP monitoring			
Mean 24-hr Systolic BP ≥ 130 mmHg	29 (64)	18 (78)	0.24
Mean 24-hr Diastolic BP ≥ 80 mmHg	24 (53)	17 (74)	0.10
Mean 24-hr awake Systolic BP ≥ 135 mmHg	26 (58)	18 (78)	0.094
Mean 24-hr awake Diastolic BP ≥ 85 mmHg	19 (42)	15 (65)	0.073
Mean 24-hr asleep Systolic BP≥ 120 mmHg	32 (71)	19 (83)	0.30
Mean 24-hr asleep Diastolic BP≥ 75 mmHg	20 (44)	16 (70)	0.050
Nocturnal variation in BP			1.00
** **Dippers	10 (22)	5 (22)	
** **Other forms of dipping (severe, reverse, non-dippers)	36 (78)	18 (78)	
Microvascular retinopathy			0.45
** **Mild	41 (89)	19 (83)	
** **Moderate	5 (11)	4 (17)	

### Arteriole and venular calibre equivalents

Retinal arteriolar calibre was reduced in subjects with a mean 24 hour ambulatory awake systolic BP ≥ 135 mm Hg (p = 0.005) **(Table 4)**. In general differences in venular calibre with different BP characteristics were less pronounced.

**Table 4:** Microvascular calibre, and BP characteristics and left ventricular hypertrophy.

Characteristic	Count	CRAE		CRVE	
		Mean ± SD	p-value	Mean ± SD	p-value
All Cases	114	134.1 ± 15.3		198.7 ± 25.4	
Clinic systolic BP					
** **< 140 mmHg	26	137.9 ± 13.9	0.154	206.5 ± 22.4	0.076
** **≥ 140 mmHg	87	133.0 ± 15.7		196.4 ± 26.0	
Ambulatory BP monitoring					
Mean 24-hour BP					
** **< 130 mmHg	32	138.1 ± 13.6	0.095	197.7 ± 24.8	0.736
** **≥ 130 mmHg	81	132.7 ± 15.8		199.5 ± 25.7	
Mean 24-hour awake BP					
** **< 135 mmHg	39	139.7 ± 12.7	0.005	200.0 ± 23.0	0.752
** **≥ 135 mmHg	74	131.3 ± 16.0		198.4 ± 26.7	
Mean 24-hour asleep systolic BP					
** **< 120 mmHg	26	136.0 ± 11.8	0.511	193.1 ± 22.9	0.182
** **≥ 120 mmHg	87	133.7 ± 16.3		200.7 ± 25.9	
Nocturnal variation in BP					
** **Dippers	28	132.8 ± 13.2	0.615	195.0 ± 18.9	0.382
** **Other forms of dipping	86	134.6 ± 16.0		199.9 ± 27.2	
LVH					
** **No	39	137.6 ± 14.1	0.014	203.0 ± 27.2	0.016
** **Yes	21	128.1 ± 13.5		185.4 ± 24.6	

CRAE-central retinal arteriole equivalent; CRVE–central retinal venous equivalent; LVH–Left ventricular hypertrophy

Retinal arteriole calibre was smaller in subjects with LVH (128.1 ± 13.5 μm) compared to those with no LVH (137.6 ± 14.1 μm) (p = 0.014). Venular calibre was also smaller in subjects with LVH (185.4 ± 24.6 μm compared to 203.0 ± 27.2 μm, p = 0.016) **(Table 4).**

## Discussion

This study suggests that retinal arteriole narrowing may be equally accurate in predicting LVH as any clinic or ambulatory BP measurement. The convenience and accuracy of microvascular calibre measurement mean that it should be investigated further for routine hypertension assessment and management. Calibre measurements have the added advantages of being inexpensive, easy to repeat, and potentially useful in monitoring BP over time. The technique is fast and allows more people to be tested and people to be tested more economically.

The presence of LVH is itself associated with an increased morbidity, including increased fatal and non-fatal cardiovascular events (myocardial infarction, heart failure, arrhythmia, stroke and sudden death) [21].

This study found that most subjects referred for ambulatory BP monitoring had an elevated clinic BP, and many were considered hypertensive on 24 hour testing criteria. All had a mild or moderate microvascular retinopathy, but mild changes were much more common than a moderate retinopathy. Microvascular retinopathy reflects end-organ damage since it correlates with cardiac ischemia, cerebral ischemia, and renal failure [22,23]. Although a moderate retinopathy was associated with an elevated mean awake systolic BP, this retinopathy occurred too infrequently to be useful clinically in identifying poorly- controlled hypertension.

The present study assessed different methods of BP measurements: clinic BP; ambulatory BP including mean 24 hour, awake and asleep systolic and diastolic BP; and abnormal dipping patterns; as well as LVH; and considered whether retinal microvascular changes reflected BP control as well as these. The results demonstrated that retinal microvascular narrowing correlated with elevated systolic BP on ambulatory BP monitoring, and with LVH; and that retinal narrowing can be used to identify individuals at greatest risk of LVH without the need for 24 hour BP monitoring.

Why was the moderate retinopathy less useful than microvascular narrowing in predicting poorly controlled hypertension? The explanation may be that the features of a moderate retinopathy are only transient, for example, haemorrhage resolves over days to weeks [24]. In contrast, the vessels narrow gradually with time, and typically take months to reverse, even if not fully, after successful BP treatment.

Can retinal arteriolar narrowing be used to assess hypertension control in the clinic? This would be difficult in a one-off examination because of the confounders associated with smaller vessel calibre including age, gender, atherosclerosis, and renal impairment [22]. In addition, coincidental inflammation, diabetes, obesity, smoking and dyslipidemia are all associated with larger calibre [25]. A complicated algorithm or a better defined normal range for different ages might be needed. On other hand it should be possible to follow an improvement in BP control with the return of vessel calibre towards normal over two or so months.

The strengths of this study were its novelty, its high recruitment rate, the unselected nature of the study subjects, and the rigour with which the retinal grading was performed. Its major weakness was that relatively few study subjects had undergone echocardiography because this was performed for clinical indications and not as an integral part of this unfunded study. The clinical indications for echocardiography were not known but about one third had known ischemic cardiac disease. It is unlikely that this skewed results because hypertension is only one contributor to cardiac ischemia together with diabetes and smoking, whereas it is a major contributor to LVH development. Although the duration of hypertension was not known, in general, study subjects all had hypertension for at least 5 years. Other weaknesses were that the study was undertaken at a single centre, and was cross-sectional and observational.

These results suggest that reduced arteriole calibre should be investigated further for assessing BP control, and whether calibre can be used to manage and monitor BP control over time. Retinal imaging may also be used to confirm hypertension in an individual with a single elevated BP measurement or indeed in a subject who is thought to be normotensive.

## Supporting information

S1 Table Deidentified data Fariya microvascular narrowing. (XLSX)
